# Comparison of the therapeutic effects of mesenchymal stem cells derived from human dental pulp (DP), adipose tissue (AD), placental amniotic membrane (PM), and umbilical cord (UC) on postmenopausal osteoporosis

**DOI:** 10.3389/fphar.2024.1349199

**Published:** 2024-03-27

**Authors:** Chuncai Li, Yincong Liu, Mingxing Deng, Jun Li, Shengqi Li, Xiaoyu Li, Yuling Zuo, Chongyang Shen, Yichao Wang

**Affiliations:** ^1^ Stem Cells Research Center, Chengdu University of Traditional Chinese Medicine, Chengdu, China; ^2^ TCM Hospital of Sichuan Province, Chengdu University of Traditional Chinese Medicine, Chengdu, China; ^3^ Sichuan Provincial Cells Tissue Bank, Chengdu, China; ^4^ Department of Thyroid Surgery, West China Hospital, Sichuan University, Chengdu, China

**Keywords:** osteoporosis, mesenchymal stem cells, Th17/Treg, macrophage polarization, immunoregulatory activity

## Abstract

**Background:** Osteoporosis is a systemic bone disease characterized by bone loss and microstructural degeneration. Recent preclinical and clinical trials have further demonstrated that the transplantation of mesenchymal stem cells (MSCs) derived from human adipose tissue (AD), dental pulp (DP), placental amniotic membrane (AM), and umbilical cord (UC) tissues can serve as an effective form of cell therapy for osteoporosis. However, MSC-mediated osteoimmunology and the ability of these cells to regulate osteoclast-osteoblast differentiation varies markedly among different types of MSCs.

**Methods:** In this study, we investigated whether transplanted allogeneic MSCs derived from AD, DP, AM, and UC tissues were able to prevent osteoporosis in an ovariectomy (OVX)-induced mouse model of osteoporosis. The homing and immunomodulatory ability of these cells as well as their effects on osteoblastogenesis and the maintenance of bone formation were compared for four types of MSCs to determine the ideal source of MSCs for the cell therapy-based treatment of OVX-induced osteoporosis. The bone formation and bone resorption ability of these four types of MSCs were analyzed using micro-computed tomography analyses and histological staining. In addition, cytokine array-based analyses of serological markers and bioluminescence imaging assays were employed to evaluate cell survival and homing efficiency. Immune regulation was determined by flow cytometer assay to reflect the mechanisms of osteoporosis treatment.

**Conclusion:** These analyses demonstrated that MSCs isolated from different tissues have the capacity to treat osteoporosis when transplanted *in vivo*. Importantly, DP-MSCs infusion was able to maintain trabecular bone mass more efficiently with corresponding improvements in trabecular bone volume, mineral density, number, and separation. Among the tested MSC types, DP-MSCs were also found to exhibit greater immunoregulatory capabilities, regulating the Th17/Treg and M1/M2 ratios. These data thus suggest that DP-MSCs may represent an effective tool for the treatment of osteoporosis.

## Introduction

Osteoporosis is characterized by the progressive loss of bone mass and skeletal microarchitecture, resulting in increased bone fragility and a higher risk of fractures (Hurley and Khosla, 1997). Women with postmenopausal osteoporosis are particularly prone to fractures, contributing to worse quality of life and imposing a substantial financial burden on affected patients. While osteoporosis is already regarded as a major public health problem in many countries (Lindsay, 1993), its incidence continues to rise with the progressive aging of the global population. It has been reported that approximately 50% of postmenopausal women worldwide are affected by osteoporosis, and the prevalence of fractures among osteoporosis patients is as high as 40% (Rachner et al., 2011). The disruption of the homeostatic balance between bone formation and bone resorption is considered to be the direct cause of osteoporosis. The relative magnitudes of bone formation and bone resorption vary at different stages of osteoporosis development, with osteoclast-mediated increases in bone resorption predominating in the early stage (Eriksen et al., 1990) whereas osteoblast-mediated reductions in bone formation are evident in the later stages (Avioli, 1976). Immunomodulation plays an important role in bone homeostasis, as the overactivation of immune responses results in the production of inflammatory cytokines that promote osteoclast differentiation and accelerate bone resorption, while also inducing osteoblast apoptosis and suppressing bone formation (Fischer and Haffner-Luntzer, 2022). Given these effects, a growing number of studies have suggested that efforts to regulate the immune system may represent a more effective means of treating osteoporosis (Yang X. et al., 2021; Wang et al., 2021).

Recently, mesenchymal stem cells (MSCs) have attracted substantial attention owing to their immunomodulatory activity and ability to undergo osteogenic differentiation such that they are thought to offer potential value as a treatment for osteoporosis (Chen et al., 2021). To date there have been many clinical trials and studies exploring the therapeutic utility of various types of MSCs in the context of osteoporosis, revealing a range of effects mediated by diverse mechanisms. Commonly used types of MSCs in these prior studies include those derived from adipose tissue (AD-MSCs), dental pulp (DP-MSCs), placental amniotic membranes (AM-MSCs), and umbilical cord tissue (UC-MSC), with these cells exhibiting varying levels of efficacy. Prior mechanistic studies have demonstrated that AD-MSCs secrete a variety of bone cell-activating factors, including hepatocyte growth factor and extracellular matrix proteins (Lee et al., 2011), while also promoting osteogenesis and inhibiting lipogenesis in osteoporotic bone marrow MSCs (BM-MSCs) through the activation of the BMP-2 and BMPR-IB signaling pathways (Ye et al., 2014). DP-MSCs attenuate bone resorption by inhibiting interferon (IFN)-γ and interleukin (IL)-17 through the enhancement of M2 macrophage polarization (Maeda et al., 2022) and promote increased bone formation by secreting RNAs that target the telomerase activity of recipient BM-MSCs (Sonoda et al., 2020). AM-MSCs can reportedly ameliorate osteoporosis by stimulating osteoclastic bone formation and inhibiting osteoclastic bone resorption (Yin et al., 2022). UC-MSC can transfer the potently pro-osteogenic CLEC11A (C-type lectin domain family 11, member A) protein to inhibit osteoclast formation and enhance the transitioning of BM-MSCs from lipogenic to osteogenic differentiation (Hu et al., 2020a). With respect to the osteogenic ability of these different cell populations, DP-MSCs have been shown to exhibit a superior osteogenic capacity but a weaker lipogenic potential than that of BM-MSCs (Kumar et al., 2018). BM-MSCs have a higher propensity for osteogenesis than AD-MSCs (Mohamed-Ahmed et al., 2018), AM-MSCs (Barlow et al., 2008), and UC-MSC (Heo et al., 2016), however invasive procedures to obtained BM-MSCs and low abundance in bone marrow (approximately 0.01%) limited its clinical application. This suggests that DP-MSCs may offer superior therapeutic efficacy in osteoporosis. However, no studies to date have compared the relative efficacy of these MSC types for the cell therapy-based treatment of osteoporosis.

To address this gap in knowledge, it is important to explore the effects of different MSC populations on osteoporotic phenotypes in order to identify the most suitable cell type for the treatment of this debilitating condition. Accordingly, this study was developed with the goal of determining which MSC type can provide the greatest benefit when used for the treatment of osteoporosis while also clarifying the key mechanisms underlying the therapeutic effects of these cells.

## Materials and methods

### Cell culture

Human AD-MSCs, DP-MSCs, AM-MSCs, and UC-MSC were provided by Sichuan Provincial Cells Tissue Bank. Cells were cultured in α-MEM (Invitrogen, Carlsbad, CA, USA) supplemented with 10% heat-inactivated FBS (Gibco, Carlsbad CA, USA), 2 mM L-glutamine (Gibco), 100 U/mL penicillin, and 100 μg/mL streptomycin (Gibco), in a 37°C, 5% CO_2_ incubator (Sanyo, Osaka, Japan). Cell growth medium was changed every 3 days. Cells were passaged with 0.125% trypsin (Gibco) at 75% confluence. To generate lines stably expressing luciferase, AD-MSCs, DP-MSCs, AM-MSCs, and UC-MSC were transduced with an appropriate virus (Lenti-EF1-Luc-Puro) and selected with 1 μg/mL puromycin for 2 weeks.

### Osteoclast differentiation of RAW264 cells

For osteoclast induction, different kinds of MSCs and RAW264.7 cells were co-cultured in a transwell insert 6-well plates, 50 ng/mL RANKL (R&D) was added to the culture medium. TRAP staining solution was added, and incubated for 40 min at 37°C. TRAP-positive cells containing three or more nuclei were considered osteoblasts.

### Animals

Eight-week-old female C57BL/6 mice were purchased from Huafukang Company (Beijing, China) and were adaptively fed for 1 week. All mice were randomly divided into six groups (n = 12 per group). Referring to previous reports (Sophocleous and Idris, 2019), mice in five groups were anesthetized via the intraperitoneal injection of sodium pentobarbital and underwent ovariectomy (OVX), while the remaining group underwent sham surgery (a small amount of fat tissue was removed from the abdominal opening, but the ovaries were not removed). These five groups of OVX mice were then subjected to different interventional conditions, including blank control treatment or treatment with AD-MSCs, DP-MSCs, AM-MSCs, or UC-MSC. At 1 week post-OVX, 1×10^6^ stem cells in 200 μL of saline were injected into the corresponding cell treatment groups via the tail vein, while blank control mice were injected with an equal amount of saline. Injections once a month for 2 months. All mice were housed in the same room with access to the same food and water. These animals were maintained at 23°C (±1°C) in a facility with 50% relative humidity and a 12 h light/dark cycle. Mice were euthanized after 2 months and appropriate tissues or organs were harvested according to the experimental requirements. All animal experiments were carried out in accordance with ethical standards and approved by the Institutional Animal Care and Use Committee of Chengdu University of Traditional Chinese Medicine (IBD2023000).

### Micro‐computed tomography (µCT) analyses

The femurs isolated from the mice were scanned following the recommendations by the American Society for Bone and Mineral Research. Briefly, dissected mouse femurs were fixed in 4% paraformaldehyde for 48 h and analyzed by high-resolution µCT (Skyscan1276, Bruker, Belgium). The scanner was set to 85 kV and 200 µA with a resolution of 10 μm, and two-thirds of each femur was scanned. A fixed threshold of 180 was used to manually contour the trabecular bone in the region of interest. For subsequent analysis, 200–500 layers below the growth plate were defined as the region of interest (ROI). The whole trabecular bone was isolated for three-dimensional reconstruction to quantitative μCT analysis.

### Histological analyses

Femurs were decalcified with 10% EDTA for 4 weeks and paraffin embedded. Femur sections were then subjected to staining with hematoxylin and eosin (H&E) and tartrate-resistant acid phosphatase (TRAP). Images were captured with a Digital triocular camera microscope (Motic, China).

### Bioluminescent imaging

Ten minutes before imaging, mice were intraperitoneally injected with 10 mL/kg of fluorescein (Cat#MX4603, MK, China) and anesthetized with inhaled isoflurane. Bioluminescent images were obtained using an *In Vivo* Imaging System (IVIS) Spectrum (PerkinElmer), and image reconstruction and analysis were performed with the IVIS imaging software (PerkinElmer).

### Cytokine antibody array

The Mouse Cytokine Array C2 (Raybiotech, USA) was used to evaluate murine serum protein content after 8 weeks cells injection, using the protocols provided by the manufacturer. Immunoblotting results were detected via chemiluminescence (Touch Imager XL, China), and the intensity of each spot was quantified using the ImageJ software.

### Flow cytometry analysis

Flow cytometry was performed on a CytoFLEX (Beckman), and data were analyzed by FlowJo software. The live cells were observed by the Fixable Viability Stain 780 (Cat#565388, BD Pharmingen). For cell-surface staining, cells were stained with BV510 Rat Anti-Mouse CD45(Cat#563891, BD Pharmingen), FITC Hamster Anti-Mouse CD3e (Cat#553061, BD Pharmingen), PE-Cy7 Rat Anti-Mouse CD4 (Cat#552775, BD Pharmingen), BV421 Rat Anti-Mouse CD25 (Cat#562606, BD Pharmingen), FITC Rat Anti-CD11b (Cat#557396, BD Pharmingen), BV421 Rat Anti-Mouse F4/80 (Cat#565411, BD Pharmingen), PE Rat Anti-Mouse CD86(Cat#561963 BD Pharmingen), Alexa Fluor 647 Rat Anti-Mouse CD206 (Cat#565250 BD Pharmingen). For intracellular staining, cells were stained with PE Rat Anti-Mouse IL-17A (Cat#561020 BD Pharmingen), Alexa Fluor 647 Rat anti-Mouse Foxp3(Cat#560402 BD Pharmingen). According to the reagent vendor’s instructions, Purified Rat Anti-Mouse CD16/CD32 is used to exclude background fluorescent signal interference, Cells fixation and permeabilization Fixation/Permeablization Kit (Cat#554714 BD Pharmingen) and Transcription Factor Buffer Set (Cat#562574 BD Pharmingen) were used for cell fixation and permeabilization.

### Statistical analysis

GraphPad Prism software (v7) was used to conduct statistical analysis (GraphPad Software). All data were expressed as the mean ± SD. Differences between the two experimental groups were determined by means of an unpaired two-tailed Student’s t-test. One-way ANOVA was used to compare more than three groups. The results were statistically significant with *p* values: ***p < 0.001; **p < 0.01; *p < 0.05.

## Results

### MSC-based cell therapy prevents OVX‐induced bone loss

**FIGURE 1:**
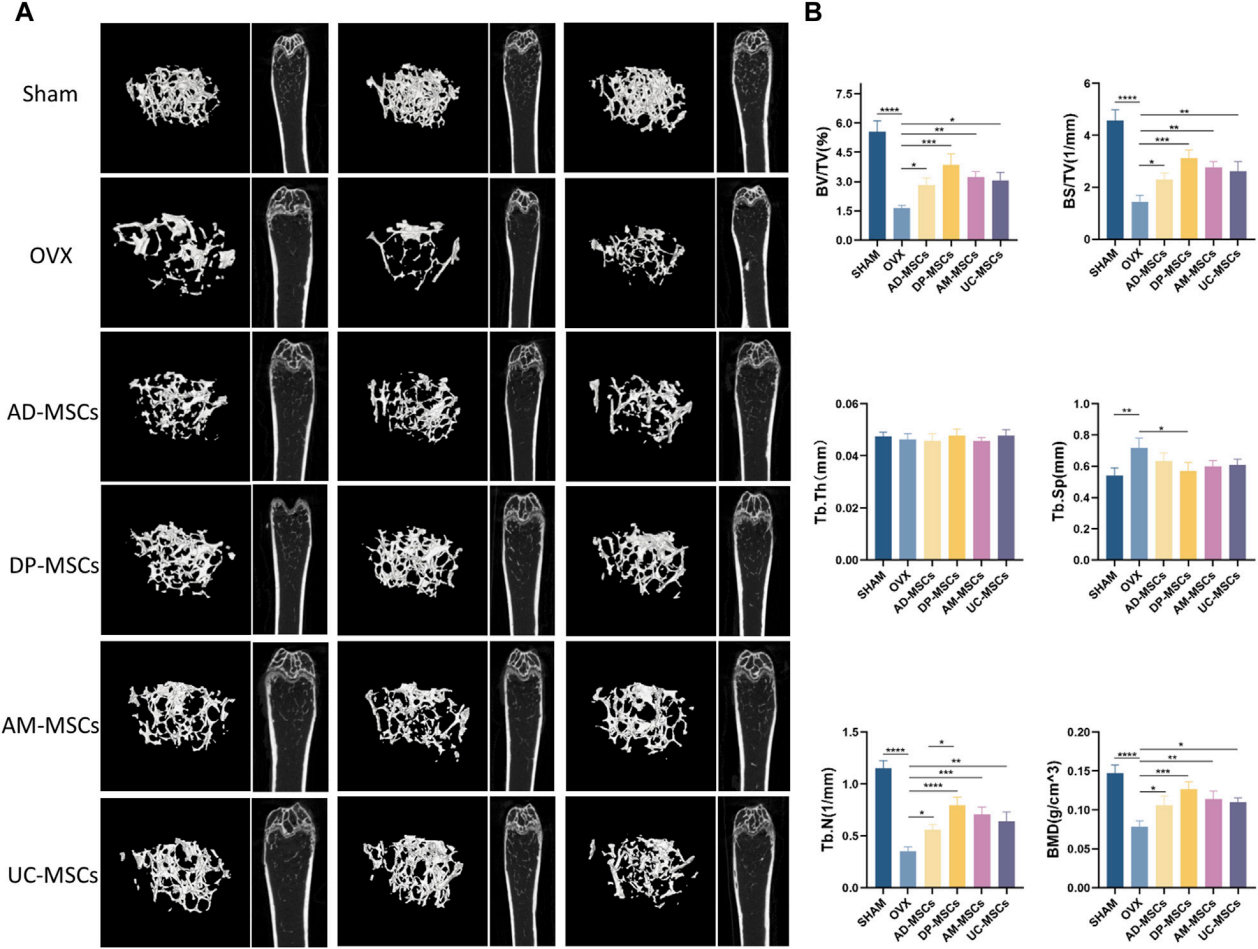
MSCs transplantation prevents bone loss in mice with osteoporosis. **(A)** Images of μCT reconstructions of distal femurs (n = 3). **(B)** Quantitative μCT analyses of the distal metaphyseal region of femurs (n = 3).

The therapeutic effects of AD-MSCs, DP-MSCs, AM-MSCs, and UC-MSC were investigated by transplanting these cells into the OVX mice via the tail vein. The mice were randomly divided into six groups (n = 12 per group). Each injection was performed 8 weeks after the induction of the OVX model. Observations were made 8 weeks after cell injection. The right femur and the distal femoral epiphysis were separated and analyzed qualitatively through µCT scanning, as this is the area most sensitive to estrogen deficiency according to previous reports (Jee and Yao, 2001). Three-dimensional imaging results showed that OVX mice had much less bone mass than normal mice. In comparison to the OVX model group, trabecular bone structure and bone mass recovery were evident in the treatment groups (Figure 1A), suggesting MSCs were able to protect against the bone loss caused by ovarian resection. However, no significant changes in bone mineral density (BMD) were observed in OVX model mice over this same period. All four types of MSCs transplantation significantly improved trabecular bone volume/total volume (BV/TV), bone surface/total volume (BS/TV), BMD, and trabecular number (Tb.N). A significant decrease in trabecular space (Tb.Sp) was evident in the DP-MSCs and AM-MSCs transplantation groups compared to the OVX model group. In contrast, cellular therapy had little effect on trabecular thickness (Tb.Th) (Figure 1B). Notably, in comparison to the other groups, the DP-MSCs group exhibited the strongest therapeutic response, highlighting the promise of this approach to treating osteoporosis (Figure 1B).

### MSC-based cell therapy reduced OVX-induced increases in bone marrow osteoclast and adipocyte counts

**FIGURE 2:**
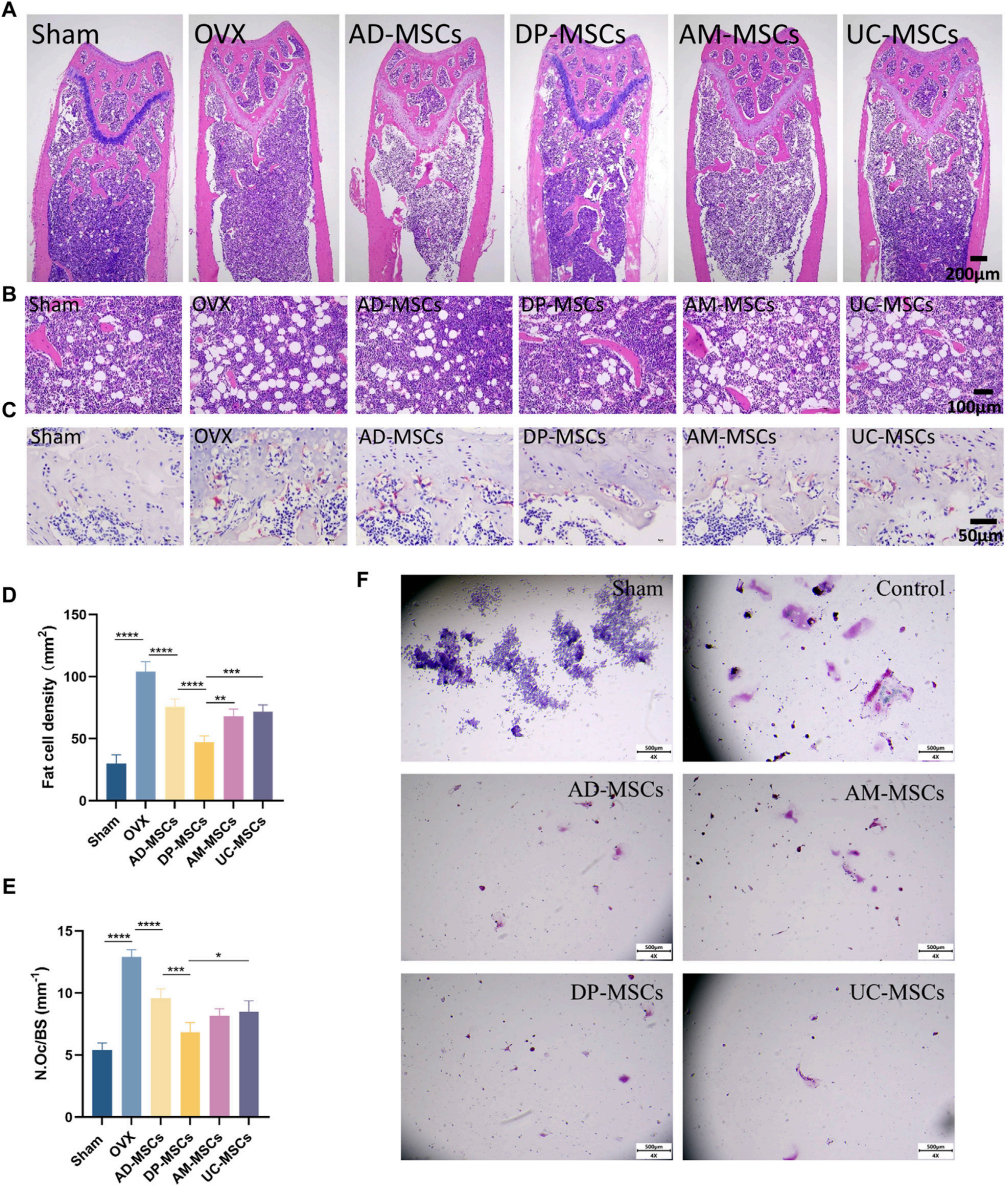
MSCs decrease the number of osteoclast and adipocytes. **(A)** Representative H&E staining of femur sections from these different MSCs treatment and control groups. **(B)** Exhibiting the marrow adipocytes. **(C)** TRAP staining of femur sections. Scale bar, 50 μm.**(D)** The numbers and area of adipocytes in the distal marrow per tissue area. **(E)** Quantification of osteoclasts in different experimental groups (n = 3). **(F)** Effect of MSC co-culture on osteoclast differentiation of RAW264 cells. Scale bar, 50 μm. Data are expressed as mean s± SD. *p < 0.05; **p < 0.001; ***p < 0.0001.

To further determine the therapeutic effect of MSCs on bone loss in OVX mice, we observed adipocytes and osteoclasts in the bone marrow cavity. H&E staining revealed that bone marrow adipocyte density increased after OVX, whereas this cell density was decreased in the AD-MSCs, DP-MSCs AM-MSCs, and UC-MSC groups compared with the OVX model group (Figures 2A, B, D), suggesting that MSCs may reduce bone loss due to OVX by reducing bone marrow adipocyte generation. TRAP staining showed that bone marrow osteoclast abundance increased after OVX, whereas there were fewer osteoclasts shown in the AD-MSCs, DP-MSCs AM-MSCs, and UC-MSC groups (Figures 2C, E), suggesting that MSCs may prevent bone loss by reducing bone marrow osteoclast production. Accordingly, DP-MSCs had the best regulatory effect on adipocytes and osteoclasts. DP-MSCs thus presented with markedly higher osteogenic ability owing to their ability to inhibit adipocyte differentiation and suppress osteoclast formation, outperforming other types of tested MSCs. These results further support the potential value of DP-MSCs as an option for the treatment of osteoporosis.

To study the impact of mesenchymal stem cells (MSCs) on the differentiation of osteoclast cells *in vitro*, we conducted transwell cultures using MSCs and RAW264.7 cells (Figure 2F). demonstrates that in the control group, osteoclasts were differentiated from RAW264.7 cells following stimulation with RANK-L, leading to a considerable increase in both the number and size of osteoclasts. Conversely, in the indirect co-culture with MSCs, the number of differentiated osteoclasts substantially decreased, and there was hardly any observed osteoclast differentiation. All four types of MSCs exhibited the ability to inhibit osteoclast differentiation, yet there were no significant differences among them.

### MSCs are short-lived *in vivo* following intravenous administration

**FIGURE 3:**
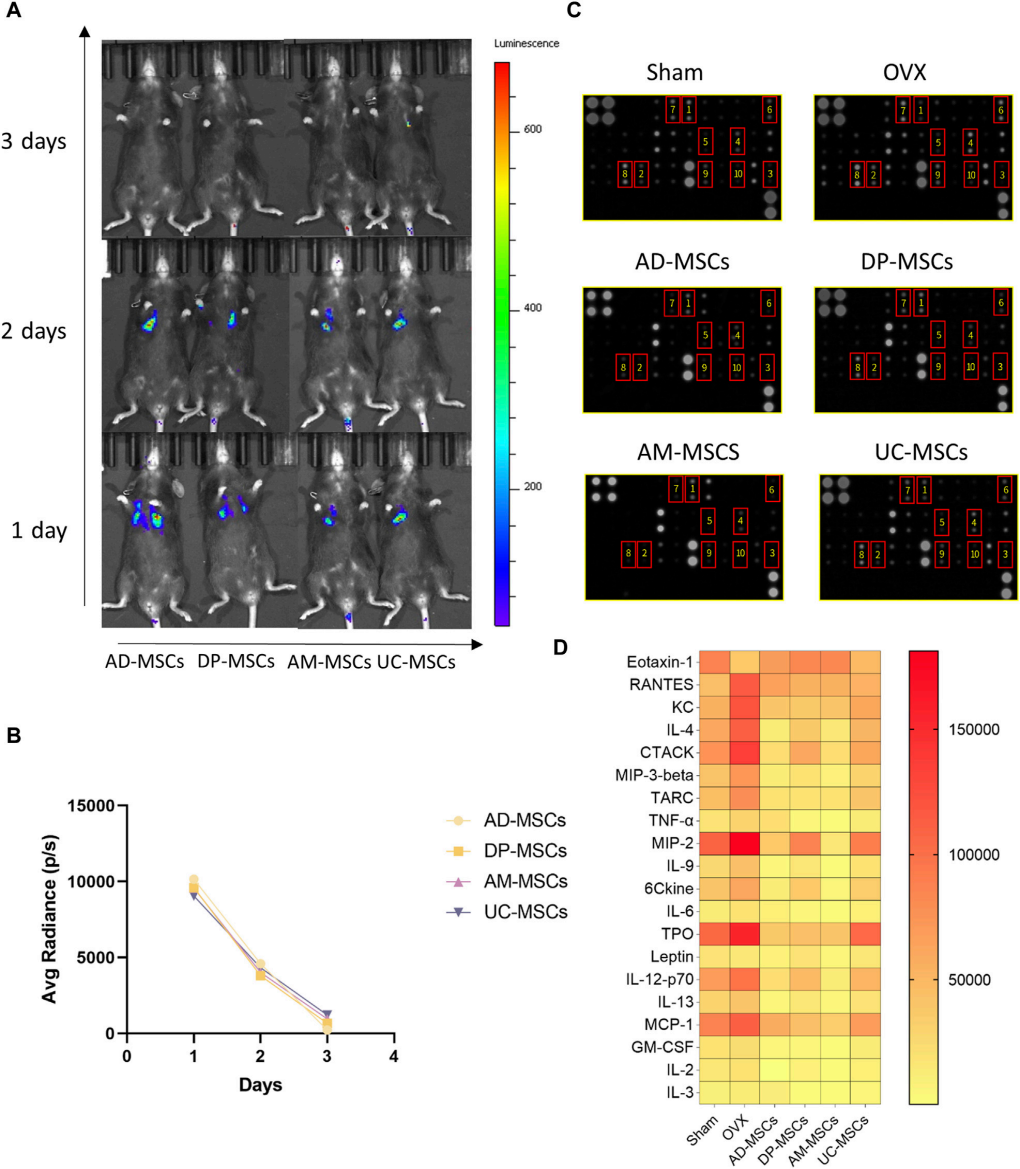
MSCs regulate peripheral serum cytokine levels through paracrine effects. **(A)** The distribution of MSCs in OVX model mice was determined via bioluminescent imaging. **(B)** The results of average radiance value analyses. **(C)** Images of the membranes used in serum cytokine antibody array analyses. Serum was separated from these different MSCs treatment and control groups. **(D)** Heatmap data demonstrating the results of comparative analyses of the same serum cytokine levels in mice in the sham, OVX, AD-MSCs, DP-MSCs, AM-MSCs, and UC-MSC groups (n = 2).

MSCs are capable of migrating to the bone marrow and playing a local role in the induction of bone formation, exerting therapeutic effects by inducing peripheral tolerance and migrating to bone marrow after *in vivo* administration (Uccelli et al., 2008). In an effort to explore the homing efficiency and survival of MSCs following systemic transplantation, we tracked these four types of MSCs transduced with luciferase genes in each treatment group to observe their *in vivo* retention and distributions. Cells were detected via bioluminescent imaging on days 1, 2, and 3 after infusion, revealing that most of these MSCs migrated to the lungs after transplantation, with the cell signal having markedly decreased by day 3 (Figure 3A). On day 1 after cell infusion, all mice exhibited comparable levels of luminescence intensity (average radiance: 1×10^4^ P/S). After 3 days, luminescence was largely absent in these animals, demonstrating that these MSCs cannot survive *in vivo* for extended periods such that their ability to improve OVX-induced osteoporosis may primarily be mediated through paracrine and immunoregulatory mechanisms (Figure 3B).

### MSCs regulate bone metabolism through paracrine effects

Given the short lifespan of MSCs following their *in vivo* delivery, paracrine effects are likely to play an important role in their therapeutic benefits. As such, we compared peripheral blood cytokine expression profiles in these different treatment groups, as cytokines can regulate bone remodeling and play a vital role in bone homeostasis (Li et al., 2020). We measured the levels of chemokines and inflammatory cytokines in serum using an antibody array, as they play important roles in the incidence of osteoporosis (Brylka and Schinke, 2019; Damani et al., 2022). Compared to the sham-operated group, the serum levels of inflammatory cytokines and chemokines were significantly altered in osteoporotic mice, including Eotaxin-1 (CCL11), RANTES (CCL5), KC (CXCL1), IL-4, CTACK (CCL27), MIP-3β (CCL19), TARC (CCL17), MIP-2 (CXCL2), TPO, IL-12-p70, and MCP-1 (CCL2), (Figures 3C, D). In contrast, MSCs-transplanted mice exhibited elevated levels of serum cytokines associated with repressing adipogenesis and activating osteogenesis as compared to OVX-induced osteoporosis model mice (Figures 3C, D). These results suggest that MSCs prevent bone loss in OVX mice through paracrine functions.

### Eotaxin-1, RANTES, and CXCL1 may be the key cytokines underlying the superior efficacy of DP-MSCs

**FIGURE 4:**
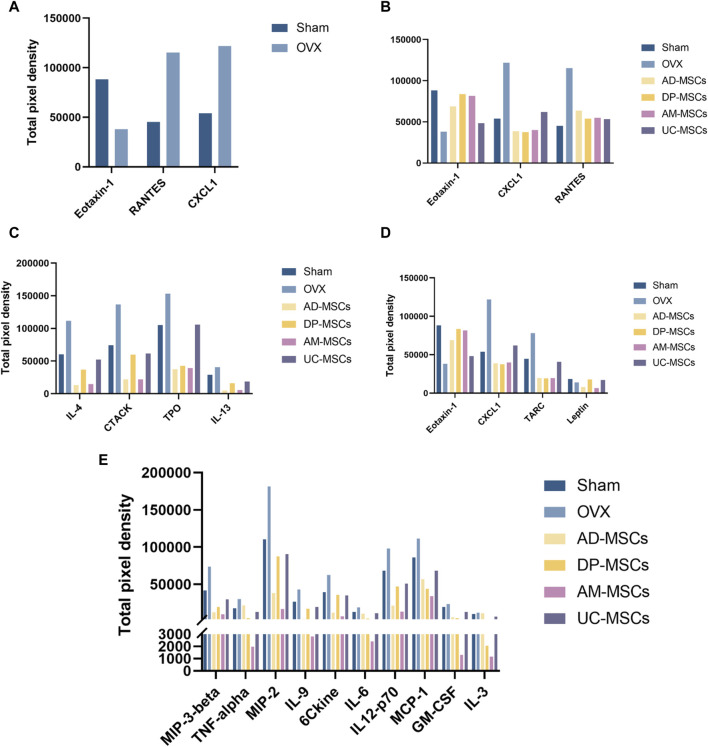
MSCs regulate serum cytokines. **(A)** Cytokines associated with significantly altered abundance when comparing the serum of normal and osteoporotic mice. **(B)** Analyses of the ability of MSCs to regulate Eotaxin-1, RANTES, and CXCL1. **(C–E)** The serum cytokines associated with the most significant improvements in the AD-MSCs **(C)**, DP-MSCs **(D)**, AM-MSCs **(E)** groups.

To explore the mechanisms underlying the paracrine effects of MSC infusion in OVX-induced osteoporosis model mice, we next compared the levels of serum cytokines related to bone remodeling in different treatment groups. The cytokines exhibiting the most significant changes were Eotaxin-1, RANTES, and CXCL1 (Figure 4A). CXCL1 and Eotaxin-1 were significantly associated with a reduction in BMD. Serum levels of CXCL1 and Eotaxin-1 were correlated with bone mass. RANTES plays a role as an inflammatory factor, associated with massive bone resorption (Lechner et al., 2018; Ahmadi et al., 2020; Korbecki et al., 2022). We then compared the effects of each type of stem cell on these three indicators and found that DP-MSCs provided a significant increase in Eotaxin-1 and dramatic decreases in the levels of CXCL1, with RANTES levels also declining to the levels observed in healthy controls (Figure 4B). Other MSCs were also associated with changes in serum cytokine levels. More specifically, AD-MSCs downregulated IL-4, CTACK, TPO, and IL-13 (Figure 4C), while DP-MSCs downregulated CXCL1 and TRAC and upregulated Eotaxin-1 and Leptin, which are also linked to bone metabolism (Figure 4D). AM-MSCs downregulated IL-17, MIP-3β, TNF-α, MIP-2, IL-9, CCL21, IL-6, IL-12-p70, MCP-1, GM-CSF, IL-3 (Figure 4E), and UC-MSCS downregulated RANTES (Figure 4B). Taken together, our findings suggest that DP-MSCs may exhibit optimal anti-osteoporotic therapeutic effects by modulating the levels of Eotaxin-1, RANTES, and CXCL1, which are associated with bone metabolism.

### DP-MSCs regulate Th17/Treg cell homeostasis and macrophage polarization

**FIGURE 5:**
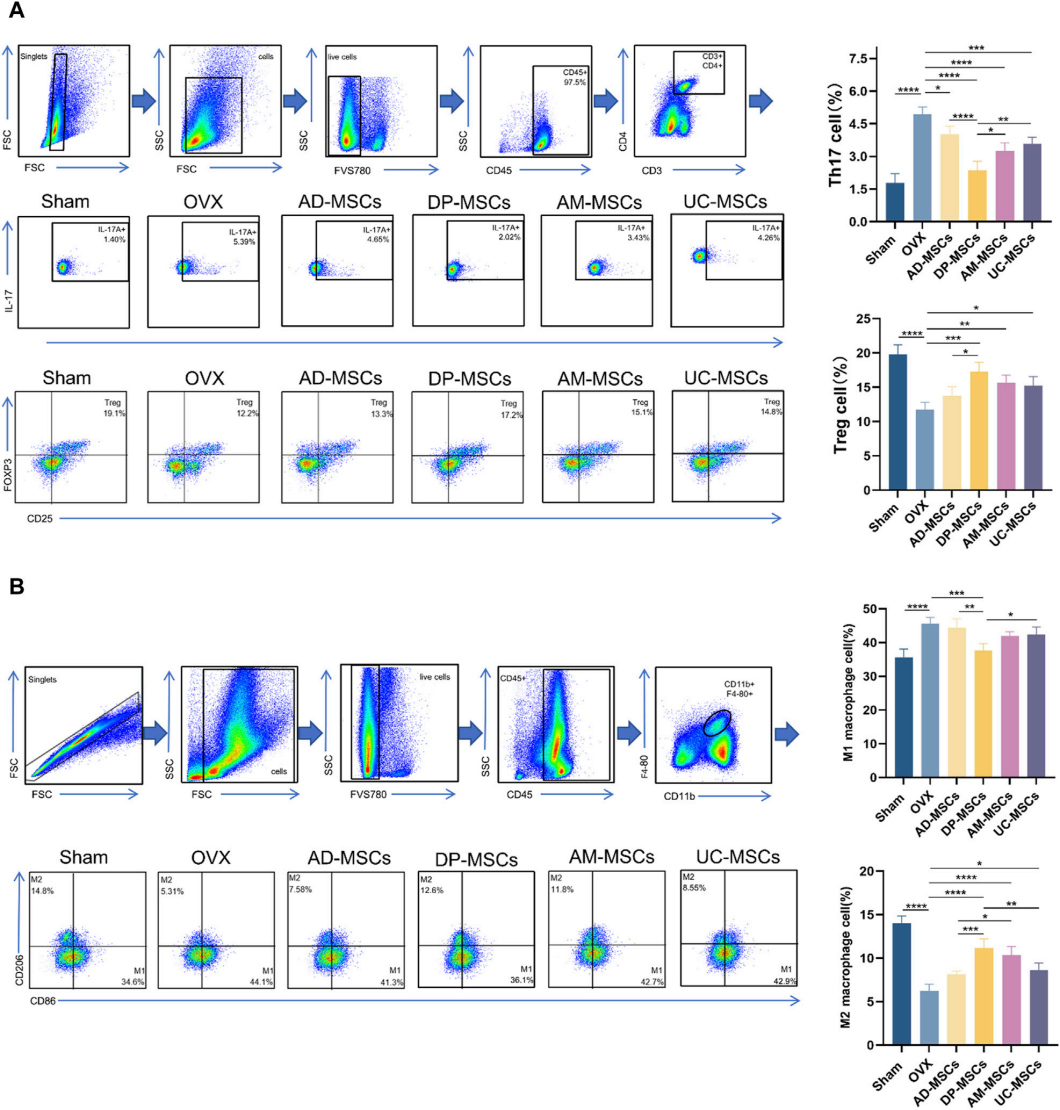
MSCs regulate splenicTh17/Treg cell balance and bone marrow macrophage polarization. **(A)** Representative flow plots and corresponding quantification of IL-17^+^ and Foxp3^+^ cell abundance in spleen (n = 4). **(B)** Representative flow plots and corresponding quantification of CD86^+^ and CD206^+^ cells in bone marrow (n = 4). Data are expressed as means ± SD. *p < 0.05; **p < 0.001; ***p < 0.0001.

MSC therapy has been extensively investigated in the context of the regulation of bone metabolism and the immune system. As such, we next assessed the ratio of splenic Th17/Treg cells and femoral macrophage polarization in our experimental mice. Th17/Treg cell homeostasis and appropriate macrophage polarization have both been shown to have a profound effect on osteoporosis (Muñoz et al., 2020; Zhu et al., 2020). Accordingly, we observed a slight decline in splenic Treg cells and an increase in Th17 cell abundance following OVX, while these changes were reversed after MSC transplantation (Figure 5A), suggesting that DP-MSCs improve OVX-induced osteoporosis through paracrine effects and immune regulation. Macrophages also participate in osteoclastogenesis by regulating the subtype M1/M2 balance and activity through cytokine signaling. MSC transplantation can also modulate the polarization of macrophages in the bone marrow. Specifically, DP-MSCs decreased the abundance of M1 macrophages and decreased the abundance of M2 macrophages more efficiently than other MSCs (Figure 5B). These results suggest that the mechanism through which MSC infusion improves OVX-induced osteoporosis entails the regulation of Th17/Treg cells homeostasis and macrophage polarization.

## Discussion

Early studies on the cell therapy-based treatment of osteoporosis have focused on how to apply MSC transplantation to improve OVX-induced osteoporosis in rodent model systems. MSCs like AD-MSCs, DP-MSCs, AM-MSCs, and UC-MSC have been widely used and their effects on immune regulation and the induction of osteogenic differentiation have been verified repeatedly. These past studies have demonstrated that mechanisms underlying MSC-based anti-osteoporotic therapy are mainly reflected by the promotion of osteoblast activity and the downregulation of osteoclast activity. MSCs have also exhibited promise as tools for the treatment of osteoporosis in many studies (Jiang et al., 2021).

Ideal candidate stem cell type for anti-osteoporotic therapy requires several characteristics, including (1) the ability to home to damaged sites to exert a local functional role, which MSCs can achieve by differentiating into bone-forming cells or acting in a paracrine manner through the secretion of cytokines to remodel the local microenvironment so as to improve bone formation; (2) immunoregulatory properties, with MSCs and immune cells being able to interact to affect the differentiation and activity of immune cells and the secretion of various immune factors; and (3) the ability to balance bone metabolism, favoring osteogenic differentiation by regulating the development of osteoclasts and osteoblasts. Recently, several studies have focused on different cell populations for the cell therapy-based treatment of osteoporosis. In particular, AD-MSCs transplantation was reported to improve bone formation and prevent OVX-induced bone loss via the secretion of various osteogenic growth factors (Jiang et al., 2021). Another study demonstrated that after transplantation, UC-MSC exhibited significant inhibitory effects on T cell proliferation, with decreases in TNF-α levels, suggesting that UC-MSC may play an immunoregulatory role. In addition, DP-MSCs have been reported to have a higher osteogenic capability and low adipogenic potential. Furthermore, Sanvoranart et al. observed that the long-term engraftment of AM-MSCs long-term was associated with significantly increased bone formation (Sanvoranart et al., 2014). However, no study to date has evaluated differences in the therapeutic potential of DP-MSCs, AD-MSCs, AM-MSCs, and UC-MSC for the treatment of OVX-induced osteoporosis. In this study, we found that MSCs isolated from different tissues exhibited varying levels of cell therapy-based efficacy.

We first evaluated the ability of four MSCs types to protect the femoral bone in OVX mice. Different types of MSCs exhibited different therapeutic effects on osteoporosis. Notably, compared with AM-MSCs, UC-MSC, and AD-MSCs, DP-MSCs exhibited superior protective effects on OVX mice, and these data indicated that the systemic infusion of DP-MSCs restored trabecular bone mass with corresponding increases in trabecular bone volume, trabecular separation, trabecular number, and BMD, although they did not prevent changes in trabecular thickness. Additional analysis revealed that DP-MSCs therapy significantly increased bone volume while decreasing marrow adipocyte and osteoclast abundance. In addition, although the transplantation of MSCs was able to protect against bone volume changes and trabecular bone loss, its effects on Tb.Th were minimal. As such, MSC transplantation may best be deployed in combination with other therapeutic modalities, such as herbal remedies (Lin et al., 2020).

Bone is a dynamic organ that undergoes continuous remodeling via coordinated synergism between osteoclasts and osteoblasts. Osteoblasts originate from MSCs in the bone marrow, and dysregulated osteoblastic differentiation of MSCs has been tied to the initiation of osteoporosis. Accordingly, transplanted MSCs rescue bone loss either by homing to the bone marrow and/or promoting osteoblastic differentiation or immunomodulatory effects. As osteogenic differentiation and immunomodulatory activity are the main mechanisms whereby MSCs can treat osteoporosis, we therefore monitored the distribution and survival of MSCs transplanted *in vitro*. Our results are consistent with previous reports that MSCs transplanted *in vivo* do not survive for extended periods and that they rarely home to the bone marrow, suggesting that paracrine effects through the secretion of certain growth factors panel with immunomodulatory properties may be the primary mechanism through which these cells function (Kong et al., 2018). In our study, we found that most engrafted MSCs were retained in the lungs, less than 50% of infused MSCs survived for 48 h, and almost none remained alive after 72 h, suggesting that MSCs in this model system mediate their effects through a paracrine role, rather than through sustained engraftment in the bone marrow. The underlying mechanism behind the limited lifespan of MSCs and its impact on their efficacy remains uncertain. Nonetheless, a study conducted by MJ Hoogduijn revealed that when MSCs were infused, they were swiftly eliminated by monocytes through phagocytosis. This process further prompted the monocytes to adopt an immunosuppressive phenotype (de Witte et al., 2018).

The loss of appropriate homeostatic balance between the bone and the immune system compartments can arise under inflammatory conditions, leading to enhanced bone loss (Fischer and Haffner-Luntzer, 2022). Immune cells can regulate bone-related cells by secreting various immune factors (Saxena et al., 2021). Given the role of the immune system in this context, it is important to clearly define the relationship between various subsets of immune cells including Th17/Treg and M1/M2 cells, the progression of osteoporosis, and the therapeutic effects of MSCs.

Macrophages are key players in the regulation of bone homeostasis. Macrophages undergo microenvironment-specific polarization, which can lead to the development of populations including classically activated (M1) and alternatively activated (M2) macrophages. Under osteoporotic conditions, macrophages display mainly a pro-inflammatory M1 phenotype and release inflammatory cytokines such as IL-1, IL-6, IL-17, and RANTES, which are closely related to increased bone resorption and osteoclast activity (Souza and Lerner, 2013; Zhang et al., 2017). Macrophage inflammatory protein-1α (MCP-1α) has been reported to induce osteoclast formation via the activation of the MEK/ERK/c-Fos pathway (Tsubaki et al., 2010). We found that MIP-2 is also likely to play a role in bone resorption. AD-MSCs (Yang CY. et al., 2021), DP-MSCs (Tian et al., 2023), AM-MSCs (Cao et al., 2020), and UC-MSC (Li et al., 2022) can all regulate macrophage polarization to ameliorate disease. However, there have been no prior comparisons of their relative ability to regulate macrophage polarization in the context of osteoporosis. Our results show that M1 macrophage abundance rose significantly following OVX in mice, while MSC transplantation was able to partially abrogate increases in these M1 macrophage levels. In contrast, transplantation of MSCs in a porcine model reportedly led to not only an increase in M2 macrophage abundance, but also to reduced levels of inflammatory cytokines such as MCP-1, TARC, CXCL1, RANTES, and CTACK. Observational studies have reported associations between levels of MCP-1 and osteoporosis, in line with its ability to regulate osteoclast differentiation and maturation (Mulholland et al., 2019). RANTES is released by human adipose tissue that induces leukocyte migration (Appay and Rowland-Jones, 2001). RANTES is overexpressed in osteoporosis (Lechner et al., 2018) and promotes osteoclast formation and bone resorption (Feng et al., 2020). CXCL1 is a CXC family chemokine that may offer value as a new predictor of early bone loss (Hu et al., 2020b). Serum CXCL1 levels are positively associated with human osteoporosis (Hardaway et al., 2015) and may cause osteoporosis by accelerating cell senescence (Korbecki et al., 2022), mediating chronic inflammation (Shi et al., 2018), and promoting the migration and maturation of osteoclast progenitors (Govey et al., 2014; Hardaway et al., 2015). TARC and CTACK mediate the migration of T cells (Hijnen et al., 2004).

IL-17 is a pro-inflammatory cytokine (Yao et al., 1995), and is regarded as a catalyst for the development of osteoporosis (Molnár et al., 2014; Zhao et al., 2016; Tang et al., 2020), acting primarily by indirectly promoting osteoclast formation (Amarasekara et al., 2018). Both Th17 and Treg cells play key roles in the maintenance of bone homeostasis (Zhu et al., 2020). Th17 cells increase osteoclast formation by promoting inflammation, whereas Tregs suppress osteoclastogenesis by suppressing the inflammatory response. MSCs can regulate the Th17/Treg balance (Zhang et al., 2021; Hua et al., 2022). Interestingly, the abundance of Th17 cells in the spleens of mice in this increased after OVX, while MSC transplantation abrogated this increase, with DP-MSCs exhibiting the greatest efficacy as compared to other tested MSC populations, indicating that these cells are the strongest inhibitors of T cell proliferation with less immunogenicity. However, UC-MSC have been reported to exhibit the poorest osteogenic potential (Li et al., 2014). We observed that splenic Treg cell abundance was markedly increased in response to DP-MSCs transplantation, suggesting that DP-MSCs exhibit superior immunomodulatory capabilities.

## Conclusion

**FIGURE 6:**
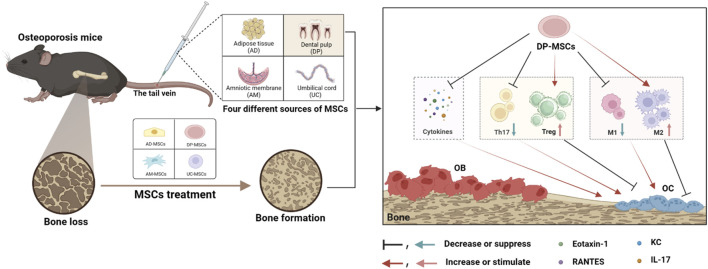
MSCs from different sources regulate the splenic Th17/Treg balance and bone marrow macrophage polarization through paracrine effects to treat osteoporosis, with DP-MSCs exhibiting the best therapeutic efficacy in this regard.

In this study, the relative performance of four different MSC types (AD-MSCs, DP-MSCs, AM-MSCs, and UC-MSC) was compared to better clarify which of these cells are best able to protect against bone loss in OVX mice through the modulation of the immune microenvironment DP-MSCs infusion restored trabecular bone mass more efficiently than other treatments, with corresponding improvements in trabecular bone volume, mineral density, number, and separation. Of these different types of MSCs, DP-MSCs were also found to exhibit a higher immunoregulatory capacity by regulating the Th17/Treg and M1/M2 ratios (Figure 6). Therefore, these results indicate that DP-MSCs may represent an effective cell population for use in the cell therapy-based treatment of osteoporosis.

## Data Availability

The raw data supporting the conclusion of this article will be made available by the authors, without undue reservation.

## Abbreviations

AD, adipose tissue; DP, dental pulp; AM, amniotic membrane; UC, umbilical cord; MSCs, mesenchymal stem cells; ECM, extracellular matrix.
